# Comparison of recombinant and urinary follicle-stimulating hormones over 2000 gonadotropin-releasing hormone antagonist cycles: a retrospective study

**DOI:** 10.1038/s41598-019-41846-2

**Published:** 2019-03-29

**Authors:** Wei Pan, Haiting Tu, Lei Jin, Cheng Hu, Jianwu Xiong, Wulin Pan, Dongyang Yu, Renjie Wang, Yuehan Li, Weiming Huang, ShuJie Liao

**Affiliations:** 1 Cancer Biology Research Center, Tongji Hospital, Tongji Medical College, Huazhong University of Science and Technology, Wuhan, Hubei 430030 PR China; 2 Economics and Management School, Wuhan University, Wuhan, 430072 China; 3 Management Science and Data Analytics Research Center, Wuhan University, Wuhan, 430072 China

## Abstract

The objective of this paper was to compare the effect of recombinant follicle-stimulating hormone (rFSH) and urinary follicle-stimulating hormone (uFSH) on pregnancy rates and live birth rates with the gonadotropin-releasing hormone **(**GnRH) antagonist protocol in China. This retrospective study was conducted from January 2014 through August 2017. Patients treated with uFSH had significantly higher levels of luteinizing hormone (3.79 mIU/ml vs. 3.09 mIU/ml) and progesterone (0.93 ng/ml vs. 1.16 ng/ml) on the day of human chorionic gonadotropin (HCG) administration, and they also had higher pregnancy rates (24.19% vs. 22.86%). There was no significant difference in the rate of live births. In the logistic regression results of the rFSH group, the pregnancy rate was positively correlated with the level of luteinizing hormone, with an odds ratio (OR) of 1.09 (95% confidence interval [CI]: 1.00–1.18; P = 0.048). In the uFSH group, the pregnancy rate was negatively correlated with the progesterone level on the day of HCG administration, with an OR of 0.47 (95% CI: 0.27–0.77; P = 0.004). Our research concluded that uFSH performed better than rFSH in terms of pregnancy rates when it was associated with the GnRH antagonist protocol. Meanwhile, no significant differences in the rate of live births were observed between the two groups.

## Introduction

One in six couples worldwide will experience at least one infertility problem during their reproductive years^1^, and the majority will benefit from assisted reproductive technology (ART). Since gonadotropin-releasing hormone (GnRH) antagonists have become available since the 1980s, and more and more researchers have paid attention to these drugs. Studies have shown that GnRH antagonists have many advantages over other GnRH analogues. For instance, some researchers have established that the GnRH antagonist protocol has also been more effective than the long GnRH agonist protocol in reducing gonadotropin consumption and the incidence of ovarian hyperstimulation syndrome (OHSS), with a shorter duration of stimulation^2–5^. Advantages of antagonists are the shorter duration of analogue treatment, the shorter duration of stimulation with follicle-stimulating hormone (FSH) and the lower risk of developing OHSS^6^. Meta-analysis results have suggested that GnRH antagonists can significantly increase the clinical pregnancy rate (CPR) and decrease the premature luteinisation rate in controlled ovarian hyperstimulation/intrauterine insemination cycles^7^.

More recently, the majority of studies on the GnRH antagonist protocol are related to progesterone, supplemented luteinizing hormone (LH), modified stimulated cycle, and so on. A study suggested that sustained FSH stimulation increases the level of progesterone during the *in vitro* fertilization (IVF) cycle. Moreover, serum progesterone prematurely appears to be interrelated to poor reproductive outcomes^8^. Research has revealed that the modified natural cycle has been shown to yield a higher live birth rate compared to the high-dose FSH cycle in the GnRH antagonist protocol^9^. Another study revealed no important difference in live birth rates with supplemental LH during IVF treatment in women who are over 35 years of age^10^. Another study confirmed that the modified FSH stimulated cycle shows a higher pregnancy rate than the normal FSH stimulated cycle in patients with a poor ovarian response^11^. However, papers have rarely compared the effect of recombinant follicle-stimulating hormone (rFSH) and urinary follicle-stimulating hormone (uFSH) on pregnancy rates and live birth rates with the GnRH antagonist protocol in a retrospective study.

Moreover, many studies have compared uFSH with rFSH with the GnRH agonist protocol regarding numbers of oocytes, pregnancy rates, live birth rates, and so on. Generally, these studies suggested that rFSH had an inferior performance in older patients than uFSH with a lower dose of FSH, but no evidence has shown that rFSH has clinical advantages for CPRs of different urinary-derived FSH gonadotropins when used with the GnRH antagonist protocol^12,13^. When choosing a GnRH analogue protocol, other factors should be considered, such as costs, patient acceptability, availability, and drug safety^13^. In addition, the manufacture of human FSH using recombinant deoxyribonucleic acid (DNA) technology (rFSH) is independent of urine collection and also guarantees a high availability of a biochemically pure FSH preparation (specific activity > 10 000 IU FSH/mg) that is free from urinary protein contaminants. It has the characteristics of high purity and low immunogenicity^13^. The uFSH pharmaceutical preparations are extracted from the urine of postmenopausal women. Therefore, the main component of these preparations is FSH, but there is also a small amount of LH^14^. The incidence of OHSS is lower after therapy with uFSH versus rFSH^14^. Further, these two drugs differ with respect to drug concentration peak times in the blood, half-life period, and prices. In summary, GnRH antagonists have unique advantages, the effects of rFSH and uFSH on assisted reproduction have been studied in other GnRH analogues, and the effects of rFSH and uFSH on assisted reproduction are insufficient studies in GnRH antagonists. Therefore, we speculated that the effects of rFSH and uFSH on assisted reproduction may be different under the GnRH antagonist protocol, which is worth studying and of great significance.

The purpose of our study was to compare the effect of rFSH and uFSH on pregnancy rates and other IVF outcomes using the logistic regression model, unlike the previous model that used the GnRH agonist protocol and mostly white individuals. By evaluating a large number of variables in common patients and selecting significant correlation variables for logistic regression model analysis, the results can be used to assess what factors ultimately determine the success of ART in general and when applied to the general population.

## Result

**Figure 1:**
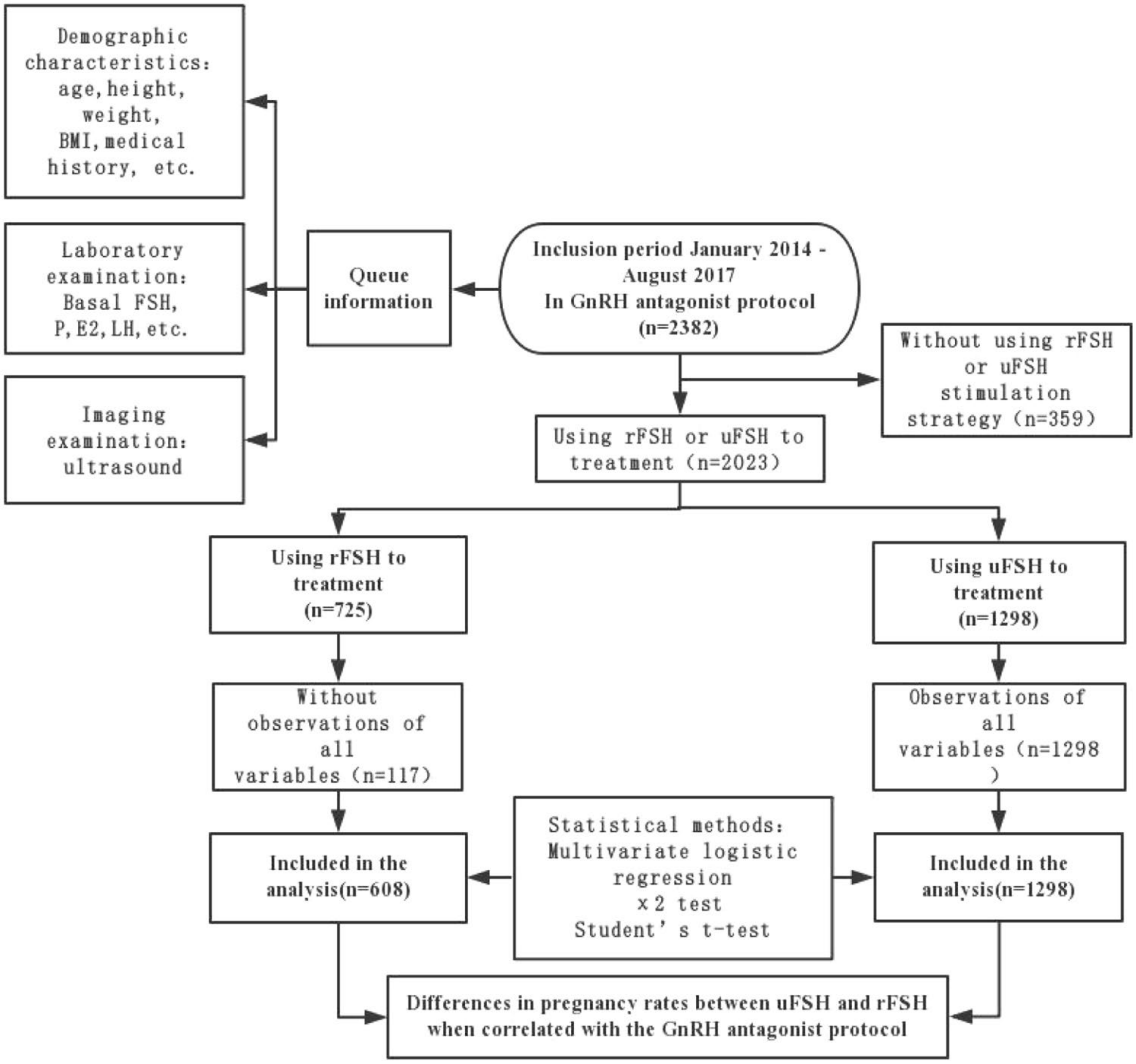
Flowchart of patient disposition throughout the study. Retrospective, Eligibility and follow-up GnRH: gonadotropin-releasing hormone rFSH: recombinant follicle-stimulating hormone uFSH: urinary follicle-stimulating hormone FSH: follicle-stimulating hormone P: progesterone E2: estradiol LH: luteinizing hormone.

A total of 476 treatments without observations of all variables were not included in the final analysis. Of the 1906 treatment cycles eligible for analysis, 32% (608 treatments) were used in the rFSH group and 1298 treatments were used in the uFSH group (Fig. 1).

**Figure 2:**
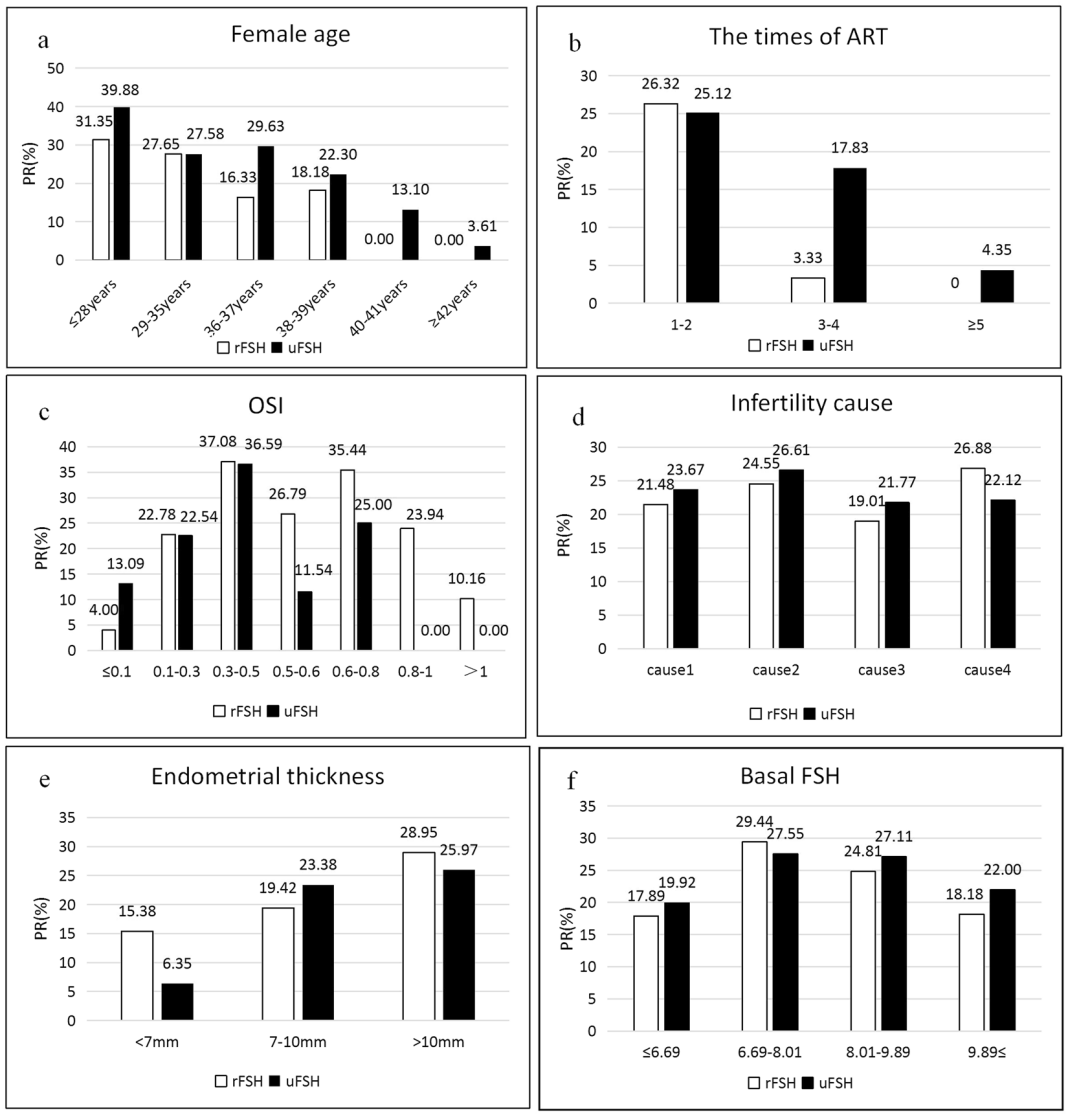
Some factors related to pregnancy rate. PR: pregnancy rate Cause1: ovulation factor Cause2: non-ovulation factor Cause3: ovulation factor plus non-ovulation factor Cause4: unexplained, etc.

Figure 2 shows the relationship between some relevant variables (e.g., age and endometrial thickness) and pregnancy rates in the rFSH and uFSH groups. According to the women’s age, we divided the patients into six groups. When women were older than 39 years and younger than 42 years, there was a significant reduction in the success rates of pregnancy compared to the previous age group (28 to 38 years old). Meanwhile, when women were older than 39 years, pregnancy success rates were zero in the rFSH group. Moreover, in any age group, the uFSH group had better pregnancy rates than the rFSH group (Fig. 2a). At the same time, patients who received ART were divided into three groups. If the patient received ART more than 5 times, pregnancy success rates were only 4.35% in the uFSH group (Fig. 2b).

The ovarian sensitivity index (OSI) measures ovarian response. Its formula is: OSI = number of oocytes retrieved/the dose of FSH^15^. When the OSI was 0.3–0.5 or 0.6–0.8, the pregnancy success rates were 37.08% and 35.44%, respectively, in the rFSH group (Fig. 2c). The cause of infertility was divided into four variables. Patients with ovulation factors plus non-ovulation factors had the lowest pregnancy rate (19.01%) in the rFSH group. However, in the uFSH group, patients with non-ovulation factors had the highest pregnancy rate (Fig. 2d). When endometrial thickness was <7 mm, the pregnancy rate was 6.35% in the uFSH group. However, the pregnancy rate with an endometrium >10 mm was 25.97% in the uFSH group (Fig. 2e).

Cycle characteristics of patients undergoing oocyte retrieval are shown in Table 1. The rFSH group was characterized by significantly higher cancelled cycles (P < 0.001), number of metaphase II oocytes (P < 0.001), number of oocytes fertilized (P < 0.001), number of oocytes retrieved (P < 0.001), oestradiol levels on the day of human chorionic gonadotropin (HCG) administration (P < 0.001), progesterone level on the day of HCG administration (P < 0.001), number of follicles on the day of HCG administration (P < 0.001) and thicker endometrium (P = 0.002) compared with the uFSH group with GnRH antagonist cycles (Table 1). The proportions of embryos transferred (P = 0.017) and the rates of clinical pregnancy (P < 0.001) were significantly lower in the rFSH group compared with the uFSH group (Table 1). In addition, there was no significant difference in live birth rates between the two groups (Table 1).

**Table 1:** Cycle characteristics of patients who occurred to oocyte retrieval in the recombinant FSH and the uFSH GnRH antagonist groups.

	rFSH(608)	uFSH(1298)	p
Embryos transferred	298 (49.01)	714 (55.01)	0.017^b^
No transferred	310 (50.99)	584 (44.99)	
**The result of ART**			
Cancelled cycles	297 (48.85)	491 (37.83)	<0.001^b^
Pregnancy failure	172 (28.29)	493 (37.98)	
Clinical pregnancy	139 (22.86)	314 (24.19)	
Live birth	124 (20.39)	253 (19.49)	0.693^b^
NO live birth	484 (79.61)	1045 (80.51)	
Number of MII oocytes	10.74 (6.50)	5.49 (3.43)	<0.001^a^
Number of oocytes fertilized	7.01 (5.00)	3.62 (2.66)	<0.001^a^
Number of oocytes retrieved	12.24 (7.19)	6.18 (3.81)	<0.001^a^
LH on day of HCG (mIU/ml)	3.09 (2.60)	3.79 (6.91)	0.001^a^
Estradiol on day of HCG (pg/ml)	3330.85 (2307.01)	1783.19 (1122.22)	<0.001^a^
Progesterone on day of HCG (ng/ml)	1.16 (0.64)	0.93 (0.48)	<0.001^a^
Endometrial thickness (mm)	11.04 (2.51)	10.66 (2.52)	0.002^a^
Number of follicles on HCG day	10.32 (5.60)	5.82 (3.00)	<0.001^a^

Data are mean(SD) or number (percentage).

^a^Two-sample t-test.

^b^Pearsonχ^2^ test.

ART: assisted reproductive technology.

MII: metaphase II.

LH: luteinizing hormone.

HCG: human chorionic gonadotropin.

There was a significant difference in low, medium, and high antral follicle count (AFCs) in the rFSH group compared with the uFSH group (P < 0.001). Regarding the number of AFCs ≥ 19, there were no differences between the two groups (Table 2).

**Table 2:** Patient and cycle characteristics by stimulation protocol and AFC category.

Antral follicle count	MII oocytes in rFSH	MII oocytes in uFSH	p
1–9(I)	6.67 (3.48)	5.17 (3.14)	<0.001^a^
10–13(II)	10.21 (4.20)	6.85 (3.77)	<0.001^a^
14–18(III)	11.75 (5.89)	9.03 (4.44)	0.017^a^
≥19(IV)	15.04 (6.98)	13.55 (5.16)	0.395^a^

Data are mean (SD).

^a^Two-sample t-test.

Comparing the patient characteristics in cycles that did and did not lead to pregnancy (Table 3), the cause of infertility was a categorical variable with three categories, and there were no significant differences in the pregnancy rates. Significant differences were found in age (P < 0.001), times of ART (P < 0.001), embryos transferred (P < 0.001), duration of infertility (P < 0.001), progesterone level on the day of HCG administration (P < 0.001), and endometrial thickness (P < 0.001) in the two groups.

**Table 3:** Baseline characteristics in cycles that did or did not result in pregnancy after treatment by antagonist protocol.

	Pregnancy (482)	No pregnancy (1519)	p
**Age (years)**			
≤28 y	149 (30.91%)	285 (18.76%)	<0.001
≤35 y	210 (43.57%)	560 (36.87%)	
≤37 y	56 (11.62%)	160 (10.53%)	
≤39 y	42 (8.71%)	159 (10.47%)	
≤41 y	19 (3.94%)	153 (10.07%)	
≥42 y	6 (1.24%)	202 (13.3%)	
**The times of ART**			
1–2	456 (94.61%)	1349 (88.81%)	<0.001
3-4	25 (5.19%)	145 (9.55%)	
≥5	1 (0.21%)	25 (1.65%)	
OSI			<0.001
≤0.1	26 (5.39%)	194 (12.81%)	
≤0.3	199 (41.29%)	698 (46.07%)	
≤0.5	172 (35.68%)	302 (19.93%)	
≤0.6	19 (3.94%)	66 (4.36%)	
≤0.8	35 (7.26%)	67 (4.42%)	
≤1	18 (3.73%)	62 (4.09%)	
≤1.2	5 (1.04%)	36 (2.38%)	
>1.2	8 (1.66%)	90 (5.94%)	
Basal FSH	8.38 (2.49)	8.68 (3.47)	0.04
Number of oocytes retrieved	8.4 (4.16)	8.47 (6.95)	0.768
Number of MII oocytes	7.57 (3.83)	7.51 (5.88)	0.788
Number of embryos transferred	1.8 (0.48)	0.69 (0.90)	<0.001
Duration of infertility (years)	3.81 (3.22)	4.57 (3.97)	<0.001
**Cause of female infertility**			
cause1	101 (20.95%)	358 (23.57%)	0.063
cause2	235 (48.76%)	641 (42.2%)	
cause3	138 (28.63%)	479 (31.53%)	
cause4	8 (1.66%)	41 (2.7%)	
Estradiol on day of HCG (pg/ml)	2215 (1218.36)	2435.07 (2001.18)	0.004
Progesterone on day of HCG (ng/ml)	0.86 (0.30)	1.06 (0.60)	<0.001
LH on day of HCG (mIU/ml)	3.25 (2.46)	3.73 (6.55)	0.017
**Endometrial thickness (mm)**			
<7	8 (1.66%)	87 (5.85%)	<0.001
7–10	160 (33.20%)	579 (38.96%)	
>10	314 (65.15%)	820 (55.18%)	
Antral follicle count	9.59 (6.96)	9.21 (7.52)	0.3
BMI (kg/m^2^)	21.9 (2.85)	22.22 (2.84)	0.035

Data are mean(SD) or number (percentage).

BMI: body mass index (kg/m^2^);

OSI: ovarian sensitivity index.

Cause1: ovulation factor.

Cause2: non-ovulation factor.

Cause3: ovulation factor plus non-ovulation factor.

Cause4: unexplained,etc.

Gn: gonadotropin.

^a^Two-sample t-test.

^b^Pearsonχ^2^ test.

By analysing the data in the previous table, we selected the significant variables for regression analysis. We finally found the best model that contains eight predictors, as shown in Table 4. The probability of pregnancy rate was significantly correlated with age (odds ratio [OR] 0.91, 95% confidence interval [CI]: 0.89–0.93; P < 0.001), embryos transferred (OR 4.88, 95% CI: 4.08–5.91; P < 0.001), endometrial thickness (OR 1.13, 95% CI: 1.08–1.19; P < 0.001), and progesterone level on the day of HCG administration (OR 0.56, 95% CI: 0.39–0.80; P = 0.002) (Table 4). The results for the rFSH group (i.e., the predictive factors for pregnancy rate after ART) along with their ORs, CIs, and P-values are presented in Table 5. This model shows that age (OR 0.91, 95% CI: 0.86–0.96; P < 0.001), embryos transferred (OR 6.90, 95% CI: 4.88–10.22; P < 0.001), endometrial thickness (OR 1.20, 95% CI: 1.10–1.31; P < 0.001), and LH level on the day of HCG administration (OR 1.09, 95% CI: 1.00–1.18; P = 0.048) differ significantly from pregnancy rate (Table 5).

**Table 4:** Logistic Regression Analysis of GnRH antagonist protocol in clinical pregnancy.

	OR (25–95%)	p
Age(years)	0.91 (0.89–0.93)	<0.001
Embryos transferred	4.88 (4.08–5.91)	<0.001
Duration of infertility (years)	0.97 (0.93–1.00)	0.071
Progesterone on day of HCG (ng/ml)	0.56 (0.39–0.80)	0.002**
Endometrial thickness (mm)	1.13 (1.08–1.19)	<0.001
Total GnRHant dose	0.93 (0.86–1.01)	0.075
LH on day of HCG (mIU/ml)	1.01 (0.98–1.03)	0.252
Cause1	*	
cause2	1.33 (0.96–1.84)	0.084
cause3	1.34 (0.94–1.91)	0.102
cause4	0.64 (0.27–1.55)	0.341

**Table 5:** Logistic Regression Analysis of GnRH antagonist protocol of recombinant FSH in clinical pregnancy.

	OR (25–95%)	p
Age (years)	0.91 (0.86–0.96)	<0.001
Embryos transferred	6.90 (4.88–10.22)	<0.001
Duration of infertility (years)	0.93 (0.85–1.01)	0.074
Progesterone on day of HCG (ng/ml)	0.74 (0.38–1.18)	0.302
Endometrial thickness (mm)	1.20 (1.10–1.31)	<0.001
Estradiol on day of HCG (pg/ml)	1.00 (1.00–1.00)	0.253
LH on day of HCG (mIU/ml)	1.09 (1.00–1.18)	0.048*
Recombinant FSH	1.00 (0.96–1.03)	0.833

We also observed that age (OR 0.91, 95% CI: 0.88–0.93; P < 0.001), number of embryos transferred (OR 4.03, 95% CI: 3.27–5.04; P < 0.001), progesterone level on the day of HCG administration (OR 0.47, 95% CI: 0.27–0.77; P = 0.004), and endometrial thickness (OR 1.11, 95% CI: 1.04–1.18; P = 0.001) differ significantly from pregnancy rate in the uFSH group (Table 6).

**Table 6:** Logistic Regression Analysis of GnRH antagonist protocol of uFSH in clinical pregnancy.

	OR (25–95%)	p
Age (years)	0.91 (0.88–0.93)	<0.001
Embryos transferred	4.03 (3.27–5.04)	<0.001
Duration of infertility (years)	0.97 (0.93–1.02)	0.233
Progesterone on day of HCG (ng/ml)	0.47 (0.27–0.77)	0.004**
Endometrial thickness (mm)	1.11 (1.04–1.18)	0.001**
Total GnRHant dose	0.90 (0.80–1.01)	0.065
Estradiol on day of HCG (pg/ml)	1.00 (1.00–1.00)	0.182
Urinary FSH	1.02 (0.99–1.05)	0.306

## Discussion

When associated with the GnRH antagonist protocol, our research revealed that uFSH was better than rFSH in terms of pregnancy rates. Moreover, we did not find a significant difference in the live birth rates between the two groups.

Our results support the conclusion of Youssef *et al*. They analysed 394 cycles with GnRH agonists, revealing that gonadotropin affects CPRs in ART cycles^11^. In our research, we analysed 1906 cycles. We selected patients using the GnRH antagonist protocol. Moreover, we also found that the uFSH group had a higher embryo transfer rate and lower cancelled cycle rate than the rFSH group, which is an interesting result. Similar findings were shown in another study^16^. Moreover, we speculate that this may be due to the use of uFSH reducing the incidence of OHSS, while rFSH has a higher rate of egg retrieval and may also increase the incidence of OHSS. However, further research is needed to confirm this speculation. In addition, there was no difference in live birth rates between the two groups. Relative studies showed that rFSH alone or in combination with LH had little impact on the outcome of a single ART cycle^17^. These different results might be due to differences in patient selection, protocol, route of administration, dose of gonadotropin, and study design.

Moreover, our results show that rFSH is more effective than uFSH in inducing metaphase II oocytes, fertilized oocytes, and follicles on the day of HCG administration. This conclusion is similar with some other studies. A meta-analysis strongly recommended that highly purified human menopausal gonadotropin (HMG) produced less oocytes with a higher total dose per cycle in ART compared with rFSH^18^. Some previous studies found that the number of oocytes retrieved was not enough to measure ovarian response. Because of the high ovarian response in women, the use of low-dose FSH could still obtain a high live birth rate^19^. For women with a low ovarian response, live birth rates did not increase when normal numbers of oocytes were obtained by prolonged stimulation or increased FSH doses^20,21^. To solve this problem, this study used OSI, which simultaneously considered the number of oocytes retrieved and FSH doses in measuring ovarian response^15^. In our study, we analysed OSI and pregnancy rates based on the work of previous researchers. We found that OSI and pregnancy rate had a normal distribution trend. Studies have shown that the incidence of OHSS increases with increases of the OSI value^15^. Therefore, we speculated that when the OSI value was low, there was low ovarian sensitivity and low pregnancy rates. When the OSI value was high, the incidence of OHSS increased and pregnancy rates decreased. Of course, this conclusion requires more studies to be proved.

By grouping the number of AFCs, we found that, as expected in the fourth group, the number of metaphase II oocytes was higher in the rFSH group compared to that in the uFSH group. It may be because rFSH is more conducive to the generation of metaphase II oocytes.

The logistic regression results of the GnRH antagonist protocol revealed that the progesterone level on the day of HCG administration was negatively correlated with the pregnancy rate. Some authors suggest that premature progesterone elevation has a negative impact on the pregnancy rate^8,22,23^. Moreover, the cause of premature progesterone elevation might be due to enhanced FSH stimulation in ART cycles^24^. In addition, the administration of HCG/LH activity would reduce this risk^25^. Several studies show that endometrial thickness is one of the independent variables predictive of clinical pregnancy^26,27^. This is in line with our findings. However, a meta-analysis including 22 studies with 10 724 *in vitro* fertilization-intracytoplasmic sperm injection (IVF-ICSI) treatment cycles revealed that the ability to determine the pregnancy rate through endometrial thickness is limited after IVF-ICSI treatment^28^.

By comparing the logistic regression results between the rFSH and uFSH groups, we found that age, number of embryos transferred, and endometrial thickness were significantly associated with pregnancy rates in both models. Studies have shown that uFSH is superior to rFSH when age is >39 years^12^. However, we found that the uFSH group had higher pregnancy rates than the rFSH group at any age, but more studies are needed to confirm this conclusion. In addition, the difference between the two groups was that the pregnancy rate was also positively correlated with the level of LH in the rFSH group. The combination of LH and rFSH leads to higher pregnancy rates^29,30^, which suggests that in order to improve the pregnancy rate, LH should be added later to the rFSH group with the GnRH antagonist protocol. However, the time and dose of LH administration needs to be further studied.

In the uFSH group, the pregnancy rate was negatively correlated with progesterone levels on the day of HCG administration, dose of GnRH antagonist, and duration of infertility. This suggests that in order to improve the pregnancy rate, relevant measures should be taken to reduce premature progesterone levels and the dose of GnRH antagonist.

This is a retrospective study. First, our data comes from clinical practice, and the results of the analysis are more in line with the actual situation faced by doctors. Second, it makes up for a gap in the literature. Past studies about two gonadotropins mainly were associated with the GnRH agonist protocol. However, our study focused on the GnRH antagonist protocol in China.

This research suggests some directions for future research, including continuing investigation to provide a clearer picture of the role of rFSH/uFSH in elderly patients, patients with a poor ovarian reserve, or both. Moreover, the cost, safety, and patient acceptability issues can also be studied. Such research would help to better answer the differences between the two drugs.

In conclusion, our research revealed that uFSH performed better than rFSH in terms of pregnancy rates when it was associated with the GnRH antagonist protocol. Meanwhile, no significant differences in the live birth rates were observed between the two groups. There are some differences in the pharmacological characteristics and prices of these two drugs. uFSH has the dual effects of FSH and LH, and the incidence of OHSS may be lower. rFSH contains a higher concentration of FSH and has a higher egg retrieval rate, which may also increase the incidence of OHSS. In addition, the price of rFSH is higher than that of uFSH. Therefore, the final therapeutic schedule was a combination of the patient’s age, duration of infertility, willingness to pay, ovarian response, willingness to accept higher cycle cancellation rates and possible risks of OHSS.

## Materials and Methods

### Study design

This study was conducted from January 2014 through August 2017 at the Reproductive Medicine Centre of Tongji Hospital, PR China. A total of 2382 infertile women who were stimulated with the GnRH antagonist protocol and had normal menstrual cycles were included in the study. Patients who underwent IVF/ICSI therapy used their own oocytes, and the embryos transferred were fresh or frozen. IVF guidelines considered ovarian, tubal, and male factors. Because the drug mainly acted on women, male factors were excluded. Other factors included endometriosis, uterine fibroids, chromosomal abnormalities, and unexplained elements. All data acquisition, management, and analyses were performed by the Data Analysis Centre of Tongji Hospital. All patients provided written informed consent to participate in the study, and we had access to information that could identify individual participants during or after data collection. According to the Institutional Review Board (IRB) of Tongji Hospital, our study was not subjected to ethics review, because all women participating in the study received routine IVF treatment in the hospital and no additional intervention or sampling was performed, as described by Yuzheng *et al*.^31^.

### Study data acquisition

All data were retrospectively collected for the purpose of investigating the clinical pregnancy outcome of patients undergoing IVF/ICSI treatment. Patients were willing and able to accomplish the protocol requirements for the duration of the study. Data were collected from all patients before ovarian stimulation. They included medical history, age, duration of infertility, types of infertility, and female cause of infertility. Meanwhile, female height and weight were collected by medical measurement and the body mass index (BMI) was calculated. Other indicators, including AFC, insemination method, number of oocytes retrieved, and doses of FSH, were also obtained by the professional medical technician.

### Study procedures

Ovarian stimulation was performed by using the GnRH antagonist protocol. After subcutaneous injection of 0.25 mg GnRH antagonist, 150 IU/day rFSH (32%) or uFSH (68%) was administered to the patient until the day of HCG administration. When a leading follicle reached 18 mm, 10000 IU HCG was administered. In two patient groups, oocyte retrieval was conducted through transvaginal ultrasound-guided aspiration 34–36 h after HCG administration. The total number of oocytes retrieved was recorded, including the number that were mature and immature.

Subsequently, fertilization methods include IVF, ICSI, IVF and ICSI, and early ICSI remediation. In addition to early ICSI remission, fertilization success was assessed 18 h after insemination. Fertilization was initially checked 6 h after insemination. If the oocytes were not fertilized, early rescue ICSI was performed immediately^32^.

Embryo transfer was performed on days 2 or 3 after oocyte retrieval, with no more than three embryos. In case the patient had OHSS, embryo transfer was cancelled. Daily vaginal progesterone was supplemented in the luteal phase, 2 applications/day, beginning on the day of oocyte retrieval until a urine pregnancy test 17 days later. The standard of positive pregnancy test is a beta HCG level of ≥5 mIU/mL; after 3 weeks, women were monitored through foetal ultrasound.

### Study outcomes

The primary outcome was clinical pregnancy, which was defined as clinical signs of pregnancy through ultrasonographic visualization of one or more gestational sacs. In addition to intrauterine pregnancy, clinical records of ectopic pregnancy are also included^33^. The other outcomes include the number of oocytes retrieved, number of metaphase II oocytes, and levels of oestradiol and progesterone.

### Statistical methods

The characteristics of patients with clinical pregnancy and without clinical pregnancy were compared. Continuous variables are expressed as the mean and standard deviation (SD), and categorical variables are expressed as percentage. Among the group comparisons, parametric tests were used for the normal distribution variables, while nonparametric tests were used the non-normal distribution variables.

Student’s t-tests were performed to evaluate the statistical relations between the subgroups. Meanwhile, the χ^2^ test was used to evaluate the significance of the proportion of the categorical variables. Multivariate logistic regression was conducted to predict the association between clinical pregnancy and potential factors such as age, embryos transferred, endometrial thickness, duration of infertility, and cause of female infertility. Because the multivariate logistic regression analysis can control the mixed effects of variables, it is widely used in most studies and proven to be effective in predicting IVF outcomes in clinical practice research^34–36^. A P-value < 0.05 was considered statistically significant. All statistical analyses were done using R language.
